# *ESR1* methylation and *ESR1* mutations in circulating tumor cells (CTCs) and paired plasma‐cfDNA of advanced breast cancer patients: A feasibility proof‐of‐concept study

**DOI:** 10.1002/1878-0261.70254

**Published:** 2026-05-27

**Authors:** Dimitra Stergiopoulou, Athina Markou, Eleonora Nicolo, Mara S Serafini, Qiang Zhang, Youbin Zhang, Lorenzo Gerratana, Andrew A. Davis, Huiping Liu, William J Gradishar, Carolina Reduzzi, Massimo Cristofanilli, Evi Lianidou

**Affiliations:** ^1^ Analysis of Circulating Tumor Cells, Laboratory of Analytical Chemistry, Department of Chemistry University of Athens Athens Greece; ^2^ Division of Hematology and Medical Oncology Weill Cornell Medicine/New York‐Presbyterian Hospital New York NY USA; ^3^ Robert H. Lurie Cancer Center of Northwestern University Feinberg School of Medicine Chicago IL USA; ^4^ Department of Medical Oncology Centro di Riferimento Oncologico di Aviano (CRO), IRCCS Aviano Italy; ^5^ Division of Oncology Washington University St. Louis MO USA

**Keywords:** breast cancer, CTCs, ctDNA, *ESR1* methylation, *ESR1* mutations, liquid biopsy

## Abstract

Endocrine resistance is a complex phenomenon, including alterations of the *ESR1* gene. The aim of this study was to simultaneously analyze *ESR1* promoter methylation and *ESR1* hotspot mutations in circulating tumor cells (CTCs) and paired plasma‐circulating tumor DNA (ctDNA) from patients with estrogen receptor‐positive (ER+) advanced breast cancer (BC). We retrospectively analyzed samples from 42 ER+ advanced BC patients characterized for CTCs and ctDNA at Northwestern University. CTCs were enumerated using the CellSearch® system, while ctDNA was analyzed with the Guardant360 NGS platform. Genomic DNA from CellSearch‐enriched CTC fractions was amplified and analyzed using the ESR1‐NAPA assay. *ESR1* methylation analysis was performed in 34 samples. *ESR1* mutations were detected in 59.5% CTC‐derived samples, a significantly higher proportion than in paired plasma ctDNA (29.6%). *ESR1* methylation was observed in 26.5% patients. Concurrent *ESR1* mutations and methylation were identified in six cases, suggesting combined genetic and epigenetic mechanisms of endocrine resistance. Overall, CTC‐derived genomic DNA showed higher sensitivity for detecting *ESR1* mutations than plasma ctDNA, supporting the potential value of CTC analysis for characterizing endocrine resistance in advanced BC.

## Graphical abstract

Circulating tumor cells (CTCs) and plasma cell‐free DNA (cfDNA) were analyzed to detect ESR1 mutations and methylation in patients with advanced breast cancer. CTC‐derived DNA showed higher sensitivity for mutation detection and revealed complementary genetic and epigenetic alterations, highlighting the added value of CTC analysis for understanding endocrine resistance.

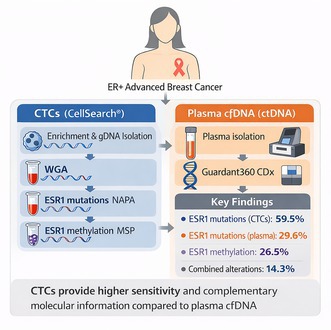

**Abbreviations**
AIs: aromatase inhibitors
BC: breast cancer
cfDNA: cell‐free DNA
CTCs: circulating tumor cells
ctDNA: circulating tumor DNA
ER: estrogen receptor
ER+: estrogen receptor‐positive
*ESR1*
: Estrogen Receptor gene
ET: endocrine therapy
gDNA: genomic DNA
HER2: human epidermal growth factor receptor 2
HR: hormone receptor
HR+: hormone receptor‐positive
IBC: inflammatory breast cancer
MBC: metastatic breast cancer
PB: Peripheral blood
PFS: progression‐free survival
SB: sodium‐bisulfite
SERDs: selective estrogen receptor degraders
SERMs: selective estrogen receptor modulators
WGA: whole‐genome amplification

## 1 Introduction

Breast cancer (BC) represents a major cause of cancer‐related mortality in women globally [1]. Metastatic disease develops in 20%–50% of patients previously diagnosed with early BC and is present at diagnosis in 6%–10% of newly diagnosed cases [2]. About 70% of metastatic breast cancer (MBC) cases are estrogen receptor (ER)‐positive/HER2‐negative (ER+/HER2‐) [3]. During the last years, there have been remarkable advances in the treatment of ER + MBC [4]. Current guidelines recommend endocrine therapy combined with CDK4/6 inhibitors as the first‐line treatment for ER+/HER2‐MBC, which has significantly improved patient outcomes. However, most ER + MBC patients ultimately develop endocrine resistance and experience disease progression during first‐line therapy [4]. Upon progression, second‐line strategies are guided by the presence of targetable alterations, including selective estrogen receptor degraders (SERDs) in the case of *ESR1* mutations. The heterogeneity of MBC requires the detection of novel biomarkers, to enable patient stratification for treatment selection and drug development, while early detection of resistance is critical for tailoring treatment strategies prior to the development of metastatic disease [5].

Liquid biopsy is a minimally invasive approach that primarily relies on the analysis of circulating tumor cells (CTCs) and circulating tumor DNA (ctDNA) [6, 7, 8]. The CellSearch®™ system is the only FDA approved platform for the detection and enumeration of CTCs in patients with MBC [9], and since then is considered as the gold standard for the detection and enumeration of CTCs for prognostication and disease monitoring in patients with metastatic breast, colon, and prostate cancer. The prognostic value of CTC enumeration in MBC patients has been confirmed in many clinical studies [10]. Molecular characterization of CTCs can reveal key signaling pathways driving metastasis, disease progression, and therapy resistance, thereby informing strategies to optimize patient management and treatment [11, 12, 13]. In the metastatic setting, somatic genetic alterations (*e.g*., mutations) can be identified in plasma cell‐free DNA (cfDNA), and the detection of druggable mutations in ctDNA, a subfraction of cfDNA, is now guiding the use of personalized therapies. Several assays have been approved by the FDA up to now, such as the Guardant 360 CDx and the Foundation One Liquid CDx tests, as companion diagnostics for the identification of genetic alterations, especially mutations in ctDNA, to guide treatment [14, 15].

Despite the efficacy of endocrine therapy in the early and MBC setting, *de novo* and acquired resistance to endocrine treatments remain a major clinical challenge [16]. One of the mechanisms of endocrine resistance in ER + MBC is the presence of *ESR1* mutations [17]. In ER + MBC, *ESR1* mutations occur in 30%–40% of patients, among which Y537S and D538G are the predominant alterations localized in the ligand‐binding domain [16]. *ESR1* mutations infrequently occur in primary tumors and typically arise in metastatic disease under the selective influence of aromatase inhibitor (AI) therapy [16]. The clinical impact of the detection of *ESR1* mutations for the management of advanced BC patients is high, as this was shown in many clinical trials till now [18, 19, 20, 21, 22, 23, 24, 25].

Our group has developed a robust and precise assay for the detection of *ESR1* hotspot mutations in EpCAM^(+)^ CTC and plasma‐ctDNA samples of ER + MBC patients [26]. Recently, using paired samples, we have reported that in CTC‐derived gDNA a higher rate of *ESR1* mutations was detected by using multiplex highly sensitive ddPCR in comparison with plasma‐cfDNA, suggesting that CTC‐derived gDNA analysis could be a highly sensitive complementary approach to the FDA‐cleared plasma‐cfDNA testing for monitoring tumor changes and guiding personalized treatments [27].

However, beyond mutations, epigenetic alterations are now established as a major hallmark of cancer [28]. For the first time, our group detected epigenetic alterations in promoters of tumor suppressor and metastasis suppressor genes in CTCs of BC patients [29, 30], and reported that the presence of *ESR1* methylation in CTCs was associated with resistance to endocrine therapy and everolimus in MBC [31].

To our knowledge, this is the first study to investigate the presence of specific hotspot *ESR1* mutations (Y537S, Y537C, Y537N, and D538G) in CTCs (extracted from CellSearch®™ cartridges) and paired plasma‐ctDNA (evaluated through Guardant360 NGS platform) of patients with ER+ advanced BC, in parallel with the *ESR1* methylation status in gDNA isolated from CTCs.

## 2 Materials and methods

**Fig. 1:**
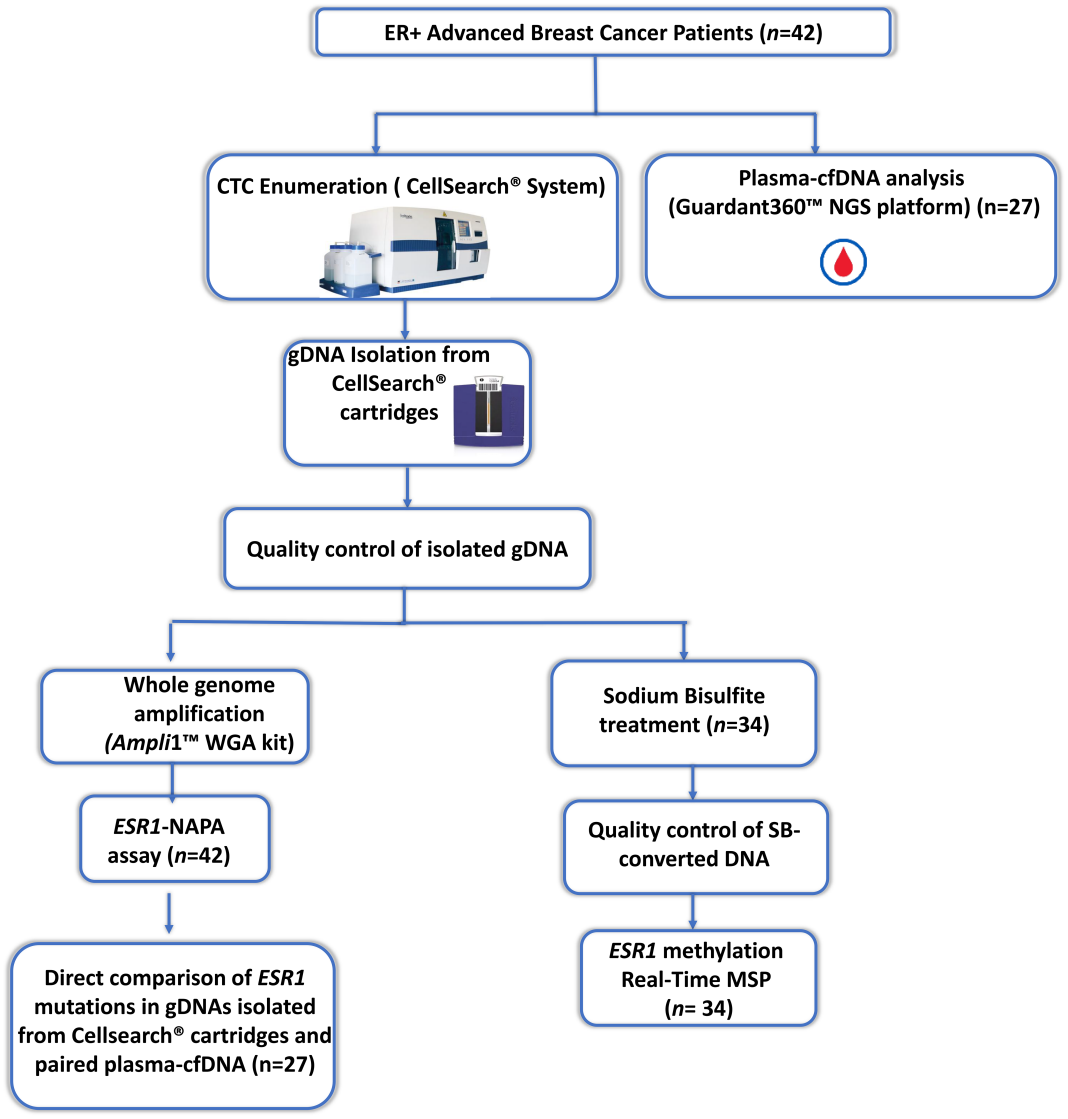
Experimental flowchart of the study.

The outline of the study is shown in Fig. 1.

### 2.1 Clinical cohort

A retrospective cohort consisting of 42 patients with ER+ advanced BC characterized for CTC at the Robert H. Lurie Comprehensive Cancer Center of Northwestern University (Chicago, IL) was considered. The study was conducted in accordance with the 1964 Declaration of Helsinki. The experiments were undertaken with the understanding and written informed consent of each participant, and the study was approved by the ethics and scientific committee of Northwestern University (IRB# NU16B06). Peripheral blood (PB) samples and other relevant specimens were collected from ER+ advanced BC patients between October 2016 and January 2021 at Northwestern University (Chicago, IL, USA). Patients underwent ctDNA analysis with the Guardant360 assay per clinical practice.

### 2.2 CTC detection and enumeration

Blood samples (7.5 mL) were collected in CellSave tubes (Menarini, Silicon Biosystems, Bologna, Italy) before starting a new treatment line. CTC enumeration was performed in the CellSearch®™ System (Menarini, Silicon Biosystems, Bologna, Italy) according to the manufacturer's instructions. Peripheral blood was collected into CellSave® preservative tubes, kept at room temperature and processed within 96 h of collection using the CellSearch®™® CTC Kit (Menarini Silicon Biosystems). For each patient, 7.5 mL of PB were analyzed. Following CTC analysis, CellSearch®™ cartridges were stored in the dark at 4 °C until gDNA isolation. Given the retrospective nature of the cohort (2016–2021), cartridge storage duration varied among samples; however, all cartridges were maintained under the same conditions (dark, 4°C).

### 2.3 Isolation of gDNA from CellSearch®™ cartridges

For the detection of *ESR1* mutations as well as the *ESR1* methylation analysis, gDNA was extracted from CellSearch®™ cartridges as previously described [29, 30, 31]. CTCs and white blood cells (WBCs) (prestained with antibody to CD45, pan‐CK, and DAPI) were aspirated from the CellSearch®™ cartridge and underwent downstream gDNA extraction using the QIAamp DNA Micro Kit (Qiagen, Germany) as previously described [29, 30, 31, 32]. Prior to downstream processing, gDNA integrity was assessed by PCR amplification of a wild‐type (WT) region in *PIK3CA* exon 20, and only quality control‐positive samples were further analyzed.

### 2.4 Quality control

gDNA recovered from CellSearch cartridges was subjected to a preanalytical quality control step to assess DNA integrity and amplifiability by PCR amplification of a constitutive WT genomic region (*PIK3CA* exon 20). This step was used exclusively for DNA QC purposes and not as a marker of CTC presence [33]. Only samples that were positive for *PIK3CA* exon 20 amplification were further processed for the mutational analysis and sodium‐bisulfite (SB) treatment for DNA conversion and downstream methylation specific PCR (MSP).

### 2.5 *ESR1* mutations

The gDNA material isolated from CellSearch® cartridges was inadequate for subsequent mutation and methylation analyses, and thus prior to *ESR1* mutation analysis, 5 μL of gDNA (0.11–0.45 ng) from all samples was subjected to whole‐genome amplification (WGA), using the Ampli1™ WGA Kit (Menarini Silicon Biosystems, Italy) according to the manufacturer's instructions. *ESR1* mutation analysis was performed using our previously developed and validated *ESR1* NAPA assay [26]. All available WGA samples were further analyzed for *ESR1* hotspot mutations [Y537S (c.1610A>C), Y537C (c.1610A>G), Y537N (c.1609T>A), and D538G (c.1613A>G)] as previously described [26]. For each *ESR1* mutation, synthetic oligonucleotide sequences were used as positive controls. Two negative controls were included in each experiment: one to control for the PCR reaction, and a second containing WT DNA as a reference template.

### 2.6 *ESR1* methylation

Since the information on methylation is lost during amplification, gDNA that was not subjected to WGA was used for DNA methylation analysis. Only samples that passed the quality control step (positive for *PIK3CA* exon 20 amplification) were processed to SB treatment using the EZ DNA Methylation Gold Kit (ZYMO Research Corp., USA), according to manufacturer's instructions. SB‐treated DNA was stored at −80°C until further use. In each SB reaction, we used the Universal Methylated Human DNA Standard (ZYMO Research Corp., USA) as fully methylated (100%) positive control and dH_2_O as negative control. Following the SB treatment, the integrity of SB‐converted DNA was assessed by a real‐time MSP for b‐actin (ACTB) as previously described [31]. The detection of *ESR1* methylation was based on our previously developed and validated real‐time MSP assay [31].

### 2.7 Statistical analysis

Statistical analysis was performed using SPSS Statistics 28.0 (IBM Corp., Armonk, NY, USA). Chi‐squared test and Cohen's kappa test were used to compare the mutational status of DNA isolated from CellSearch®™ cartridges with those of the paired plasma‐ctDNA samples.

## 3 Results

### 3.1 Clinical samples and patient characteristics

Forty‐two samples were collected from ER+ advanced BC patients: All patients included in this cohort had hormone receptor‐positive (HR+) tumors and had previously received endocrine therapy, 10 of whom were HER2‐positive. All patients were treated and characterized for CTCs at Northwestern University (Chicago, IL). CTC enumeration was performed using the CellSearch®™ system (Menarini, Silicon Biosystems, Bologna, Italy). The analysis of plasma‐cfDNA was performed using the commercially available Guardant 360 assay (Guardant Health, Inc., Redwood City, CA). Patient characteristics are shown in Table 1.

**Table 1:** Clinicopathological characteristics of 42 ER+ advanced breast cancer (BC) patients. Patients have subsequently been divided into two categories: those with mutated *ESR1* in circulating tumor cells (CTCs), as defined by whole‐genome amplification (WGA); those with methylated *ESR1* in CTCs.

Patients	All (*n* = 42) N (%)	*ESR1* mut in CTCs (*n* = 25/42) N (59.52%)	*ESR1* meth in CTCs (*n* = 9/34) N (26.5%)
Histotype			
Ductal	25 (59.6%)	18 (72.0%)	7 (77.8%)
Lobular	8 (19.0%)	2 (8.0%)	1 (11.1%)
Other	1 (2.4%)	1 (4.0%)	1 (11.1%)
NA*	8 (19.0%)	4 (16.0%)	0 (0%)
Receptor status			
HR+/HER2‐	32 (76.2%)	18 (72.0%)	5 (55.6%)
HR+/HER2+	10 (23.8%)	7 (28.0%)	4 (44.4%)
IBC			
Yes	12 (28.6%)	8 (32.0%)	2 (22.2%)
No	29 (69.0%)	17 (68.0%)	7 (77.8%)
NA	1 (2.4%)		0 (0%)
Stage at blood collection			
III	6 (14.3%)	5 (20.0%)	3 (33.3%)
IV	36 (85.7%)	20 (80.0%)	6 (66.7%)
Treatment line (for Stage IV)			
< 2	9 (25.0%)	5 (25.0%)	0 (0%)
≥ 2	27 (75.0%)	15 (75.0%)	6 (100%)
Previous ET			
Yes	32 (76.2%)	19 (76.0%)	5 (55.6%)
No	10 (23.8%)	6 (24.0%)	4 (44.4%)

* Not available.

### 3.2 Detection of *ESR1* mutations in gDNA isolated from CellSearch®™ cartridges

**Fig. 2:**
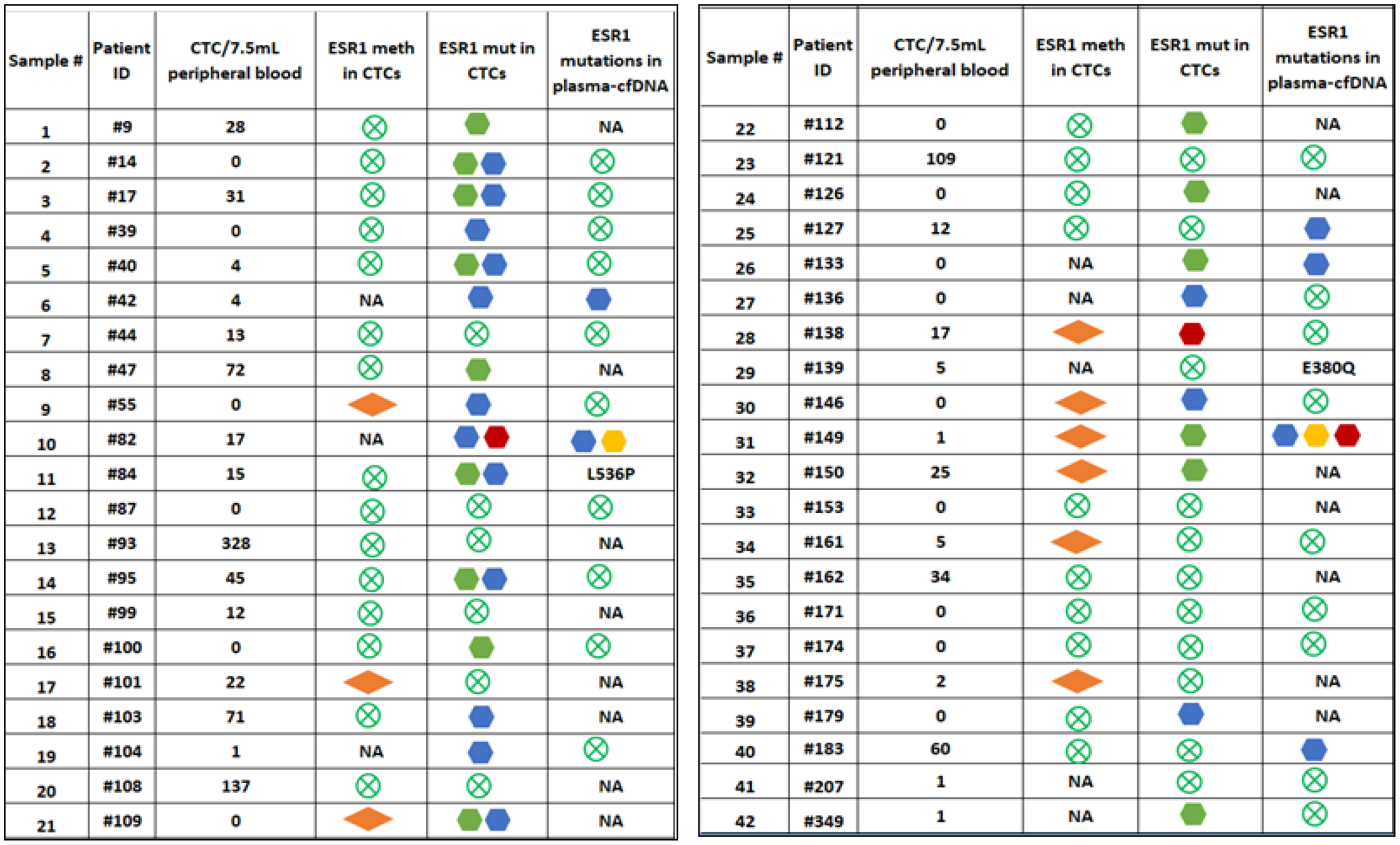
Mutation and methylation status of DNA samples isolated from CellSearch® cartridges and the mutational status of *ESR1* mutations in paired plasma‐circulating tumor DNA (ctDNA) samples of advanced breast cancer patients. 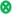, unmethylated or wild‐type; 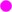, ESR1 methylated; 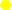, Y537C; 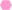, Y537S; 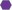, Y537N; 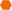, D538G; NA, not analyzed.

Following quality control assessments, all gDNA samples isolated from CellSearch®™ cartridges were deemed of sufficient quality for downstream mutational analyses. *ESR1* mutations were identified in 25 out of 42 (59.5%) gDNA samples derived from CTCs (Table 1). The most frequently observed mutations in CTCs were Y537C and D538G, each detected in 15 of 42 samples (35.7%). The Y537N variant was detected in 2 of 42 CTC‐derived gDNA samples (4.8%), whereas the Y537S mutation was not identified in any of the analyzed CTC‐derived gDNA samples (Fig. 2). *ESR1* mutations were more frequently observed in patients who had undergone two or more prior lines of therapy. Among this cohort, the average progression‐free survival (PFS) for patients with at least one *ESR1* mutation was 10.4 months, compared with 9.6 months in patients without detectable *ESR1* mutations.

Notably, 7 out of 42 patients (16.7%) harbored multiple *ESR1* mutations. Of these, six patients (P#14, P#17, P#40, P#84, P#95, and P#109) exhibited concurrent Y537C and D538G mutations, while one patient (P#82) demonstrated co‐occurrence of Y537N and D538G mutations (Fig. 2).

As illustrated in Fig. 2, CellSearch®™ analysis identified 15 patients as negative for CTCs. In four of these cases (P#87, P#153, P#171, and P#174), neither *ESR1* mutations nor *ESR1* methylation was detected in the gDNA isolated from the CellSearch®™ cartridges. Conversely, *ESR1* mutations were detected in 11 of these 15 CTC‐negative (according to CellSearch®™) patients. This might be due to the fact that CTCs might be present in the sample but not being detected using the CellSearch®™ criteria, such as CTCs lacking epithelial markers or CTCs expressing both epithelial and leukocyte markers [34]. It is interesting to note that this was not relevant to the number of CTCs, as in patients with high CTC numbers *ESR1* mutations were not detected (P#93:328CTCs/7.5 mL, P#108:137CTCs/7.5 mL, P#121:109CTCs/7.5 mL, and P#183:60CTCs/7.5 mL).

### 3.3 Direct comparison of *ESR1* mutations in gDNA isolated from CellSearch®™ cartridges and paired plasma‐cfDNA

For 27 patients, matched plasma‐derived cfDNA samples were available and analyzed for the presence of *ESR1* mutations (Fig. 1) using the FDA‐cleared Guardant360 assay (Guardant Health, Inc., Redwood City, CA) per clinical practice. This plasma‐ctDNA analysis was performed in close temporal proximity to the CellSearch®™‐based CTC assay, with a median interval of 0 days between sample collections (range: 0–43 days). *ESR1* mutations—specifically Y537S, Y537N, D538G, L536P, and E380Q—were detected in 8 out of 27 (29,6%) plasma‐cfDNA samples.

Subsequently, we conducted a direct comparison of *ESR1* mutation status between gDNA isolated from CTCs captured on CellSearch®™ cartridges and the matched plasma‐cfDNA samples. Overall, concordance for the presence of at least one *ESR1* mutation between CTC‐derived gDNA and matched plasma cfDNA was very low, only in 12 of 27 cases (44.4%) (Table 2). The results of this direct comparison clearly indicate a higher detection rate of *ESR1* mutations in CellSearch®™‐enriched CTC‐derived gDNAs (17/27 cases, 63.0%) compared with matched plasma‐cfDNA samples (8/27 cases, 29.6%), indicating that CTC‐derived gDNA may offer enhanced sensitivity for detecting *ESR1* mutations in ER+ advanced BC patients. These results are in accordance with our very recent findings, using a different group of patients, a different CTC enrichment protocol and a completely different *ESR1* detection assay based on ddPCR [27].

**Table 2:** Direct comparison between gDNA isolated from circulating tumor cells (CTCs) (CellSearch® cartridges) and paired plasma‐ctDNA samples for the presence of *ESR1* mutations.

*N* = 27		gDNA isolated from CTCs (CellSearch® cartridges)		
		+	−	Total
Plasma‐ctDNA	+	5	3	8
	−	12	7	19
	Total	17	10	27
Concordance	12/27 (44.4%) (*k* = −0.005 Cohen's kappa test, *P* = 0.651)			

‘+’: positive, at least one *ESR1* mutation was detected; ‘−’: negative, no *ESR1* mutations were detected.

Notably, 12 patients exhibited *ESR1* mutations in CTC‐derived gDNA that were not detected in the corresponding plasma‐cfDNA samples. More specifically, *ESR1* mutations were detected only in CTC‐derived gDNA but not in corresponding plasma‐cfDNA in P#14, P#17, P#39, P#40, P#55, P#95, P#100, P#104, P#126, P#136, P#138, and P#146 (Fig. 2). There were only three cases where *ESR1* mutations were detected only in plasma‐cfDNA but not in corresponding CTC‐derived gDNA (P#127, P#139, and P#183). In patient P#149, multiple *ESR1* mutations (Y537S, Y537N, and D538G) were detected in plasma‐cfDNA; however, only the Y537C mutation was identified in the paired CTC‐derived gDNA (Fig. 2). There were only five cases where CTC‐derived gDNA and paired plasma‐cfDNA were both positive for *ESR1* mutations (Table 2). However, only in one of these five cases (P#42) the same mutation, D538G, was identified, while in all other cases discordant *ESR1* mutations were detected.

### 3.4 Analysis of *ESR1* methylation in gDNA isolated from CellSearch®™ cartridges

Based on quality control assessment, 34 gDNA samples isolated from bulk CTCs enriched using the CellSearch® system were of sufficient quality for downstream *ESR1* methylation analysis. *ESR1* promoter methylation was detected in 9 out of 34 (26.5%) HR+ patient samples (Fig. 2).

Notably, concurrent *ESR1* methylation and *ESR1* point mutations were identified in six patients (P#55, P#109, P#138, P#146, P#149, and P#150) (Fig. 2), indicating potential coexistence of epigenetic and genetic mechanisms of resistance. Among the 27 patients for whom both CTC‐derived gDNA and plasma‐cfDNA were available, only one patient (P#149) exhibited a triple‐positive profile: *ESR1* mutations in plasma cfDNA, *ESR1* mutations in CTC‐derived gDNA, and *ESR1* promoter methylation in CTC‐derived gDNA. This patient had previously received multiple lines of endocrine therapy (including tamoxifen and letrozole), and palbociclib. It is also interesting to note that in three patients identified as negative for CTCs by CellSearch®™ analysis, *ESR1* methylation was detected (P#55, P#109, and P#146). In all of these patients, the D538G *ESR1* mutation was also detected in CTC‐derived gDNA.

## 4 Discussion

To our knowledge, this is the first study to explore the presence of specific hotspot *ESR1* mutations (Y537S, Y537C, Y537N, and D538G) in CTCs (extracted from CellSearch®™ cartridges) and paired plasma‐ctDNA (evaluated through Guardant360™ NGS platform) of patients with ER+ advanced BC, in parallel with the *ESR1* methylation status in gDNA isolated from CTCs, using highly sensitive and analytically validated methodologies.

Among the most clinically significant mechanisms of acquired endocrine resistance in HR+ BC is the emergence of activating mutations in the *ESR1* gene, primarily located within its ligand‐binding domain. These mutations—most commonly Y537S, Y537C, Y537N, and D538G—arise under the selective pressure of AI therapy and are associated with sustained, estrogen‐independent activation of the ER signaling pathway. The clinical relevance of *ESR1* mutations has been demonstrated in pivotal trials, such as PADA‐1 and EMERALD, leading to the approval of SERDs like elacestrant and the development of corresponding companion diagnostic assays [19, 20, 21]. PADA‐1 revealed that the presence of *ESR1* mutations significantly decreased the median PFS in first‐line treatment with an AI and the CDK4/6 inhibitor, palbociclib [18]. Detection of a rising baseline *ESR1* mutation led to a clinically and statistically improved PFS when switching to fulvestrant and palbociclib in comparison with continuing with AI and palbociclib treatment [19]. The EMERALD trial showed that patients with ER+/HER2‐ advanced/MBC and detectable *ESR1* mutations that progressed on prior endocrine and CDK4/6 inhibitor therapy and received elacestrant had a statistically significantly prolonged PFS compared with those who received standard of care ET [20]. The results of this trial led to FDA approval of elacestrant as targeted therapy for ER+/HER2‐advanced/metastatic BC patients with detectable *ESR1* mutations in plasma‐cfDNA samples [21]. In parallel, the FDA approved the Guardant360 CDx assay as a companion diagnostic device to identify patients with BC for treatment with elacestrant [21]. Gerratana et al investigated the association of *ESR1*/*PIK3CA* codon variants in ctDNA with clinical characteristics and pathway classification for MBC patients [22]. In an updated exploratory analysis of the PALOMA‐3 study, *ESR1* mutations were found to be prognostic for overall survival (OS) and were highly associated with shorter OS [23]. In another study, the presence of *ESR1* mutations was shown exclusively in distant but not local recurrences in five independent BC cohorts and *in vivo* experiments showed that *ESR1*‐mutant cells were associated with larger multicellular CTC clusters with increased compactness compared with *ESR1* WT CTCs [24]. Very recently, results of the SERENA‐6 clinical trial have shown that in patients with ER‐positive, HER2‐negative advanced BC with an *ESR1* mutation that emerged during treatment, those who were switched to camizestrant with continuation of a CDK4/6 inhibitor during first‐line therapy had significantly longer PFS than those who maintained the AI combination [25].

Several studies have emphasized the value of assessing *ESR1* mutations in CTCs at both bulk and single‐cell resolution [35, 36, 37, 38, 39, 40, 41]. In a prospective cohort of 55 women with HR+ BC, *ESR1* mutations were predictive of shorter PFS on AI therapy but not on non‐AI regimens [38]. Compared with *ESR1* mutant cases, AI‐resistant CTCs with WT *ESR1* showed an elevated ER‐coactivator RNA signature, consistent with their predicted response to second‐line hormonal therapies [38]. Additionally, CTCs harboring WT *ESR1* yet resistant to AIs showed upregulation of ER coactivator–associated gene signatures, potentially identifying tumors responsive to second‐line hormonal agents [37].

In this study, we assessed the presence of hotspot *ESR1* mutations and promoter methylation in CTCs and matched cfDNA from patients with ER+ advanced BC. *ESR1* mutations were more frequently detected in gDNA isolated from CellSearch®™ enriched CTC fractions when compared to paired plasma‐cfDNA in this cohort under the specific assay conditions. Among mutation‐positive CTC cases, Y537C and D538G were the most prevalent variants, consistent with previous reports [42, 43]. Our findings highlight the enhanced sensitivity of CTC‐based molecular analysis for detecting rare or low‐frequency clones, and are in agreement to our previous study that demonstrated a higher detection rate of *ESR1* mutations in CTC‐derived gDNA compared with paired cfDNA using the same ddPCR protocol [27]. However, our results are in contrast to the findings reported by Beije et al who reported that the sensitivity for detecting *ESR1* mutations by digital PCR in CellSearch®™‐enriched CTC fractions was lower than that for cfDNA [39]. This may be explained by the fact that in that study only two *ESR1* hotspot mutations (D538G and Y537S/N/C) were evaluated in CTC‐enriched DNA using digital PCR [39]. A particularly interesting observation was the detection of *ESR1* mutations in CellSearch®™ enriched CTC fractions from patients who had no enumerated CTCs using conventional CellSearch®™ criteria. This discrepancy may reflect phenotypic plasticity of CTCs undergoing EMT, which reduces expression of epithelial markers, such as cytokeratins and EpCAM, resulting in under‐detection by CellSearch®™ [32, 34]. Nonetheless, such CTCs may still be captured and characterized at the molecular level, as we have previously reported [32]. In previous studies, we analyzed gDNA extracted from bulk CTCs and single CTCs enriched through CellSearch® system for the detection of mutation and methylation markers [31, 32]. Due to the qualitative nature of the *ESR1*‐NAPA assay and the use of WGA, *ESR1* mutation analysis was not intended for quantitative interpretation (e.g., VAF estimation), but rather for highly specific detection of tumor‐derived *ESR1* alterations in CTC‐enriched fractions.

The emergence of *ESR1* mutations in cfDNA was associated with clinicopathological features and treatments in the adjuvant and metastatic settings; it was reported that 26% of *ESR1*‐positive patients with ER+/HER2‐ BC had more than one *ESR1* mutation suggesting polyclonal disease [44]. In another study, the heterogeneity and clinical importance of *ESR1* mutations in ctDNA samples from ER + MBC patients receiving fulvestrant was evaluated revealing that among patients with any detectable *ESR1* mutation, 22.8% had two mutations and 17.5% had more than two mutations [45].

Recently, there is a lot of interest for *ESR1* mutation testing, as a companion diagnostic for specific treatments. Beyond elacestrant, it was shown that camizestrant, a next‐generation oral SERD in combination with a cyclin‐dependent kinase (CDK) 4/6 inhibitor (palbociclib, ribociclib, or abemaciclib) demonstrated a highly statistically significant and clinically meaningful improvement in the primary endpoint of PFS over fulvestrant (also a SERD) in ER+/HER2‐ advanced BC [46]. Recent results from the Phase 3 EMBER‐3 study of imlunestrant, an investigational, oral SERD, in patients with ER+/HER2‐advanced BC, whose disease progressed on a prior AI, with or without a CDK4/6 inhibitor, have shown a statistically significant and clinically meaningful improvement in PFS as monotherapy in patients with an *ESR1* mutation versus standard of care endocrine therapy, reducing the risk of disease progression or death by 38% [47].

According to our findings, combined analysis of CTC‐derived gDNA and paired plasma‐cfDNA revealed a very high positivity rate (20/27, 70.1%) for *ESR1* mutations in ER+ advanced BC patients. Direct comparison of the presence of at least one *ESR1* mutation between the gDNA isolated from CTCs and the paired plasma‐cfDNA showed 44.4% (12/27) concordance. In 12 cases, *ESR1* mutations were detected only in CTC‐derived gDNA but not in plasma‐cfDNA. These patients, according to the guidelines, are not eligible for treatment with elacestrant. The relatively low concordance observed between *ESR1* mutation detection in CTC‐derived gDNA and matched plasma cfDNA is consistent with our previous findings, in which paired samples analyzed concurrently with the same highly sensitive assay (ddPCR) also showed limited agreement (27). This supports the notion that CTCs and cfDNA interrogate distinct, yet complementary, facets of tumor heterogeneity, and dynamics, rather than reflecting technical inconsistencies between workflows. Regarding the observed discordance in *ESR1* mutation status between CTCs and plasma‐cfDNA, several nonmutually exclusive explanations should be considered. First, tumor heterogeneity may result in different subclonal populations contributing to CTCs versus circulating DNA, with certain *ESR1*‐mutant clones preferentially detected in one liquid biopsy component but not the other. Second, differences in biological origin and kinetics between CTCs and cfDNA—such as variations in DNA shedding, clearance rates, and tumor burden—may influence detectability at a given time point. Third, very low analyte levels, which are typical for both CTC‐derived gDNA and cfDNA in many patients, inherently limit concordance even when sensitive assays are employed.

In parallel, analysis of *ESR1* promoter methylation in the same samples revealed hypermethylation in 26.5% of CTC‐derived gDNA samples. In six patients, *ESR1* promoter methylation co‐occurred with activating mutations, suggesting that genetic and epigenetic alterations may coexist and synergistically contribute to endocrine resistance. These results are consistent with recent data showing significantly lower *ESR1* promoter methylation in *ESR1‐*mutant cases, possibly reflecting suppression of methylation by transcriptionally active mutant ER [48]. Interestingly, *ESR1* promoter methylation was only observed in patients who had received multiple lines of prior endocrine therapy, further supporting its role as a late event in the evolution of endocrine resistance.

Notwithstanding the novelty of the results, the study presents several limitations. First, this is a retrospective analysis on a relatively small cohort of patients. In addition, because this is an exploratory study, the population did not have strict inclusion criteria, resulting in a very heterogeneous cohort. Because of this, even though the study shows the feasibility of *ESR1* analysis on CTCs and the possibility to increase alteration detection, no conclusions can be made on their clinical significance or treatment implications. Future studies should be conducted to validate our findings in larger cohorts and further investigate the predictive and prognostic significance of *ESR1* alteration detection on CTCs. Another methodological limitation of the present study relates to the nature of the material retained within CellSearch cartridges. In addition to intact, enumerated CTCs, cartridges may also contain tumor‐derived debris, apoptotic cells, or fragmented tumor DNA, which can contribute to downstream molecular signals even in samples classified as CTC‐negative by enumeration. Furthermore, the preanalytical DNA quality control step, based on amplification of a constitutive WT genomic region, assesses overall DNA integrity and amplifiability but does not confirm the presence or proportion of tumor‐derived DNA. As a result, samples with few or no detectable CTCs may still pass QC due to leukocyte‐derived DNA. These factors should be considered when interpreting molecular findings derived from CellSearch‐enriched fractions, particularly in the context of low CTC counts.

The discrepancy observed between CellSearch CTC enumeration and *ESR1* mutation detection further supports the concept of pronounced CTC heterogeneity. While CTC enumeration remains a robust and clinically validated prognostic biomarker, molecular characterization provides orthogonal information related to tumor biology that cannot be inferred from cell counts alone. The detection of tumor‐specific molecular alterations in CellSearch‐negative samples and their absence in samples with high CTC counts suggests that rare or phenotypically distinct CTC subpopulations may carry disproportionate molecular relevance. As this study is a proof‐of‐concept and not designed to assess clinical predictive value, these findings should be considered hypothesis‐generating and warrant validation in larger, clinically annotated cohorts.

## 5 Conclusions

In conclusion, although exploratory, the present study shows intriguing results on the genetic and epigenetic characterization of CTCs. In the present cohort and under the specific experimental and analytical conditions employed, *ESR1* mutations were more frequently detected in CTC‐derived gDNA than in matched plasma‐cfDNA and are in accordance with our recent report using ddPCR, even if they are based on a different CTC enrichment system, a different *ESR1* mutation detection method and an independent patient cohort. Our findings indicate that CTC analysis might be a very sensitive approach to evaluate endocrine resistance and tumor heterogeneity and complements ctDNA analysis. Further studies are needed to define the predictive impact of these features where CTCs will be directly compared with the paired plasma‐ctDNA using the same biomarkers and harmonized detection platforms.

## Conflict of interest

The authors declare no conflicts of interest.

## Author contributions

MC and EL conceptualized the study. DS, AM, EN, MSS, and YZ developed the methodology. DS, AM, and EL validated the study. DS, AM, EN, and MSS conducted the formal analysis. DS, AM, EN, MSS, QZ, LG, AAD, CR, HL, and WJG performed the investigation. EL, MC, and WJG provided the resources. DS, AM, EN, MSS, LG, and CR curated the data. DS, AM, CR, EN, and EL wrote the original draft. DS, AM, EN, MSS, QZ, YZ, LG, AAD, HL, WJG, CR, MC, and EL reviewed and edited the manuscript. CR, MC, and EL supervised the project. EL managed the project administration. MC and EL acquired the funding. All authors reviewed and approved the final version of the manuscript.

## Data Availability

Data supporting this study's findings can be obtained from the corresponding author upon request, but are not publicly available due to ethical considerations.
